# Prevalence and Management of Hypertension in a Tertiary Care Hospital in Amalapuram, India: A Cross-Sectional Analysis

**DOI:** 10.7759/cureus.102676

**Published:** 2026-01-30

**Authors:** Manchala Mohith, Karre Bujji

**Affiliations:** 1 Cardiac Surgery, Konaseema Institute of Medical Sciences and Research Foundation, Amalapuram, IND; 2 Physiology, Konaseema Institute of Medical Sciences and Research Foundation, Amalapuram, IND

**Keywords:** cross-sectional study, hypertension, management, physical activity, prevalence, socioeconomic factors

## Abstract

Background

Hypertension is a major global health issue and a leading contributor to morbidity and mortality. It disproportionately affects low- and middle-income countries. Behavioral and lifestyle-related factors play a major role in raising blood pressure. Understanding local prevalence patterns and identifying modifiable risk factors are essential for developing effective prevention and control strategies.

Methods

A cross-sectional study was conducted at a tertiary care center. Using consecutive sampling, a total of 240 participants were recruited. Data were collected using validated questionnaire forms, and statistical analysis was performed using SPSS version 24 (IBM Corp., Armonk, NY, US).

Results

The overall prevalence of hypertension in the study population was 93 (38.8%), with a marked difference between men (53 (46.09%)) and women (40 (32%)). Hypertension showed significant associations with key risk factors, including body mass index (BMI), physical activity, and socioeconomic status (p < 0.05). A substantial proportion of hypertensive individuals reported limited daily physical activity, with 68 (73%) engaging in less than 15 minutes of activity per day, while 69 (74%) reported no regular exercise.

Conclusion

The study demonstrates a high prevalence of hypertension with strong associations to modifiable lifestyle and socioeconomic factors. Targeted preventive strategies emphasizing physical activity, weight management, and health education are essential to reduce the burden of hypertension.

## Introduction

Hypertension remains one of the most significant global public health challenges [1]. According to the World Health Organization (WHO), the mortality rate from hypertension has increased, now constituting over 12.8% of global fatalities [2]. The number of hypertensive adults in the WHO Western Pacific region in 2019 compared to 1990 has risen from 144 million to 346 million [3]. Approximately 1.13 billion people have hypertension worldwide [4], contributing substantially to the burden of cardiovascular, cerebrovascular, and renal diseases, often leading to premature death [3,5]. Urban populations tend to show higher rates than rural counterparts [4], reflecting lifestyle and environmental differences [6].

Hypertension is a major non-communicable disease [7], affecting nearly 1.13 billion adults worldwide [8]. In India, approximately one in three adults suffers from elevated blood pressure [9], contributing heavily to morbidity and mortality [10]. The multifactorial etiology of hypertension includes genetic predisposition, dietary habits, obesity, stress, and sedentary lifestyles [11]. Urbanization and changing lifestyles have further accelerated its prevalence [6]. Effective management requires a combination of medical treatment, behavioral modification, and health education to promote early detection and adherence to therapy [12].

This study aims to determine the prevalence of hypertension and explore its associated factors among patients attending a tertiary care center in Amalapuram, emphasizing sociodemographic [13] and lifestyle determinants. Insights from this research may guide targeted interventions for improved prevention and management.

## Materials and methods

This hospital-based cross-sectional study was conducted at KIMS & RF (Konaseema Institute of Medical Sciences and Research Foundation), Andhra Pradesh. The calculated sample size was 236, rounded to 240. Participants aged over 18 years attending the outpatient department were included after obtaining informed consent. Those with severe illness or below 18 years of age were excluded.

After obtaining ethical approval from the Institutional Ethical Committee (IEC/PR/2021-11/10.09.2024), data were collected using a structured and pretested questionnaire capturing sociodemographic details, medical history, lifestyle habits (diet, smoking, alcohol, and physical activity), and treatment adherence (Appendix). Blood pressure was measured using a sphygmomanometer.

The study duration was three months. Data were analyzed. Descriptive statistics summarized participant characteristics, while chi-squared tests and logistic regression identified significant associations between independent variables and hypertension.

## Results

Out of 240 participants, 114 (47.5%) were aged 40-60 years, 86 (35.8%) were 20-40 years, and 40 (16.7%) were 60-80 years. Men constituted 121 (50.4%), and women 119 (49.6%). The socioeconomic breakdown showed that 36 (15%) belonged to the upper class, 48 (20%) to the upper middle, 96 (40%) to the lower middle, and 60 (25%) to the lower class categories.

Regarding educational status, 23 (9.58%) were illiterate, while 22 (9.1%) were professionals. Body mass index (BMI) distribution indicated 98 (40.3%) overweight and 50 (20.8%) obese participants. Blood pressure readings categorized participants as non-hypertensive (147 (61.2%)), previously diagnosed (55 (22.9%)), and newly detected hypertensives (38 (15.3%)). Only 55 (22.9%) reported regular antihypertensive medication use. Physical inactivity was prevalent, with 94 (39.1%) reporting no exercise, while 110 (45.8%) exercised less than 15 minutes per day. All the data and the outcomes of various parameters we monitored are shown in Table 1.

**Table 1:** Different types of variables along with their categories, frequencies, and percentages.

Variable	Category	Frequency (f)	Percentage
Age	20-40 years	86	35.8%
	40-60 years	114	47.5%
	60-80 years	40	16.7%
Gender	Male	121	50.4%
	Female	119	49.6%
Socioeconomic status	Upper class	36	15%
	Upper middle class	48	20%
	Lower middle class	96	40%
	Lower class	60	25%
Level of education	Illiterate	23	9.58%
	Primary	49	20.4%
	Secondary	61	25.4%
	Under-graduates	47	19.5%
	Graduates	25	10.4%
	Post-graduates	13	5.4%
	Professional	22	9.17%
Blood pressure	<90/60 mmHg	39	16.2%
	90-120/60-80 mmHg	54	22.5%
	121-139/80-89 mmHg	93	38.7%
	>140/90	54	22.5%
History of hypertension	Not hypertensive	147	61.2%
	Previously hypertensive	55	22.9%
	Newly diagnosed	38	15.3%
Duration of hypertension	No previous history	147	61.2%
	1-5 years	38	15.8%
	5-10 years	27	11.2%
	>10 years	28	11.6%
Medication history	Yes	55	22.9%
	No	185	77.0%
BMI (body mass index)	Underweight	38	15.8%
	Normal	54	22.5%
	Overweight	98	40.3%
	Obese	50	20.8%
Comorbidities	Yes	42	17.2%
	No	198	82.4%
History of alcohol consumption	Yes	30	12.5%
	No	210	87.5%
History of smoking	Yes	35	14.5%
	No	205	85.4%
Physical exercise	Yes	146	60.8%
	No	94	39.1%
Duration of exercise	<15 min/day	110	45.8%
	15-30 min/day	103	42.9%
	>30 min/day	27	11.2%

A statistically significant correlation was found between hypertension and BMI, physical activity, and socioeconomic class (p < 0.05), indicating the influence of lifestyle and social conditions on blood pressure levels.

## Discussion

The prevalence of hypertension in this study was 38.8%, higher than some national estimates [6,14] but comparable to other tertiary care-based studies [14]. This suggests a significant burden of hypertension in the population, particularly in tertiary care settings [15]. The high prevalence rate underscores the need for urgent public health attention and targeted interventions.

Men had higher hypertension rates than women, likely due to lifestyle factors such as smoking, alcohol consumption, and occupational stress [5]. These findings are consistent with existing literature highlighting the role of lifestyle in hypertension development. Physical inactivity was a major risk factor, with participants engaging in limited or no exercise exhibiting substantially higher hypertension rates [13,15].

Regular aerobic activity reduces blood pressure by improving vascular elasticity and metabolic balance. This underscores the importance of promoting physical activity as a preventive measure [16]. Obesity, measured through BMI, showed a strong association with hypertension [12], consistent with global research linking excessive body weight to increased vascular resistance [17]. Weight management strategies should be a key component of hypertension prevention and management programs [18].

Socioeconomic status was another critical determinant, with individuals from lower-income groups showing higher prevalence. This is likely due to limited healthcare access and unhealthy dietary habits. Targeted interventions addressing these barriers are essential. Community-based health education and screening can help identify high-risk individuals and promote early detection and treatment.

Public health strategies focusing on early detection, counselling, and affordability of care can substantially mitigate hypertension-related morbidity. By implementing these strategies, we can reduce the burden of hypertension and improve cardiovascular health outcomes [13].

Limitations of the study

The primary limitation of this study is its hospital-based sampling from a selected rural area, which may limit the generalizability of the findings. Furthermore, the management strategy was presented in a broad conceptual manner rather than as detailed methodological procedures. In addition, a detailed quantitative assessment of dietary intake was not included.

## Conclusions

The population exhibits a notably high prevalence of hypertension, driven primarily by modifiable risk factors such as physical inactivity, excess body weight, and socioeconomic disparities. These findings underscore the urgent need for targeted preventive strategies tailored to address these specific risk factors and reduce the overall burden of hypertension in this community.

Recommendations for prevention and control are to effectively mitigate the growing prevalence of hypertension; a multifaceted approach is necessary. Lifestyle modification programs should be prioritized, emphasizing regular physical activity, weight management, and reduced salt intake. Simultaneously, socioeconomic empowerment initiatives and improvements in healthcare access can play a pivotal role in disease control. Collaborative efforts between healthcare providers, policymakers, and local communities are essential to implement these strategies effectively and reduce the hypertension burden.

## Appendices

Data collection proforma for the study on hypertension prevalence and management

Section 1: Demographic information*

1. Age:

2. Sex:

3. Socioeconomic status:

4. Education:

Section 2: Hypertension status

1. History of hypertension:

2. What was your blood pressure reading at diagnosis?*

3. Duration of hypertension:

4. Medication history:

Section 3: Lifestyle habits*

1. Do you smoke? Yes/No

2. Do you consume alcohol? Yes/No

3. Do you exercise regularly? Yes/No

4. Duration of exercise:

5. Comorbidities:

Section 4: Physical measurements*

1. Height:

2. Weight:

3. Waist circumference:

4. Blood pressure:
